# Antiangiogenic properties of selected ruthenium(III) complexes that are nitric oxide scavengers

**DOI:** 10.1038/sj.bjc.6600906

**Published:** 2003-04-29

**Authors:** L Morbidelli, S Donnini, S Filippi, L Messori, F Piccioli, P Orioli, G Sava, M Ziche

**Affiliations:** 1 Department of Molecular Biology, University of Siena, Via Aldo Moro 2, 53100 Siena, Italy; 2 Department of Pharmacology, University of Florence, Viale Pieraccini 6, 50139 Florence, Italy; 3 Department of Chemistry, University of Florence, Via della Lastruccia 3, 50019 Sesto Fiorentino, Florence, Italy; 4 Callerio Foundation, Institute of Biological Research, Via A. Fleming 22-31, 34127 Trieste, Italy

**Keywords:** nitric oxide, ruthenium(III), angiogenesis, endothelial cells, migration, proliferation

## Abstract

The nitric oxide synthase (NOS) pathway has been clearly demonstrated to regulate angiogenesis. Increased levels of NO correlate with tumour growth and spreading in different experimental and human cancers. Drugs interfering with the NOS pathway may be useful in angiogenesis-dependent tumours. The aim of this study was to pharmacologically characterise certain ruthenium-based compounds, namely NAMI-A, KP1339, and RuEDTA, as potential NO scavengers to be used as antiangiogenic/antitumour agents. NAMI-A, KP1339 and RuEDTA were able to bind tightly and inactivate free NO in solution. Formation of ruthenium–NO adducts was documented by electronic absorption, FT-IR spectroscopy and ^1^H-NMR. Pretreatment of rabbit aorta rings with NAMI-A, KP1339 or RuEDTA reduced endothelium-dependent vasorelaxation elicited by acetylcholine. This effect was reversed by 8-Br-cGMP. The key steps of angiogenesis, endothelial cell proliferation and migration stimulated by vascular endothelial growth factor (VEGF) or NO donor drugs, were blocked by NAMI-A, KP1339 and RuEDTA, these compounds being devoid of any cytotoxic activity. When tested *in vivo*, NAMI-A inhibited angiogenesis induced by VEGF. It is likely that the antitumour properties previously observed for ruthenium-based NO scavengers, such as NAMI-A, are related to their NO-related antiangiogenic properties.

Nitric oxide (NO) is an important signalling molecule that acts in many tissues to regulate different physiological and pathological processes. We demonstrated that NO stimulates angiogenesis and mediates the effect of a number of angiogenic molecules (Ziche *et al*, 1994,^1997^; Parenti *et al*, 2001). In human tumours, NO plays an important role in tumour growth and progression (Wink *et al*, 1998), as also evidenced by the increased expression of nitric oxide synthase (NOS) found in a variety of tumours (Thomsen *et al*, 1994; Cobbs *et al*, 1995; Gallo *et al*, 1998; Klotz *et al*, 1998; Feng *et al*, 2002). Nitric oxide has been shown to be important for maintaining the vasodilator tone of tumours by regulating tumour blood flow (Fukumura *et al*, 1997), and is an active mediator of tumour angiogenesis (Gallo *et al*, 1998; Jadeski and Lala, 1999; Jadeski *et al*, 2000), intimately linked with cancer cell growth and metastasis. Other mechanisms responsible for stimulation of tumour progression and spreading by tumour-derived NO are stimulation of tumour cell invasiveness Orucevic *et al*, 1999; Jadeski *et al*, 2000) as well as increased expression of angiogenic factors (Morbidelli *et al*, 2001; Feng *et al*, 2002).

The availability of drugs able to specifically target NOS or inactivate free NO in tumours can be regarded as a new alternative therapeutic approach to inhibit tumour angiogenesis. Systemic NOS inhibition through L-arginine derivatives in experimental tumour models was demonstrated to be antitumoral, by reducing tumour volume and increasing tumour necrosis (Thomsen *et al*, 1997; Jadeski and Lala, 1999; Swaroop *et al*, 2000). However, an issue associated with NOS inhibitors is their lack of selectivity for the three isoforms of NOS (Garvey *et al*, 1997; Cobb, 1999; Cheshire, 2001). In the case of NO scavengers, the selectivity is based on the rate of reaction with NO, which is dependent on the concentration of NO and the scavenger. Ruthenium compounds, designed as NO scavengers, work by rapidly and efficiently reacting with NO (Bottomley, 1978; Fricker *et al*, 1997; Mosi *et al*, 2002, patent IPN: WO95/05814). These compounds have been shown to exhibit pharmacological activity *in vitro* and in a number of *in vivo* models for a variety of diseases including cancer (Clarke, 2002 and references therein). Furthermore, an angiogenesis inhibitory activity has been reported for NAMI-A (imidazolium *trans*-imidazole dimethylsulphoxide tetrachloro ruthenate, ImH[*trans*-RuCl4(DMSO)Im) (Sanna *et al*, 2002; Vacca *et al*, 2002), a novel ruthenium(III)-based compound developed for selectively treating solid tumour metastases (Sava and Bergamo, 1999).

The aim of the present study was to characterise the pharmacological properties of representative ruthenium(III) complexes on isolated aortic ring preparations and to test their efficacy in inhibiting NO-dependent angiogenesis both *in vitro* and *in vivo*. Specifically, we wanted to ascertain whether the NO scavenging ability of these ruthenium(III) complexes and the related interference with the angiogenic process might be the basis of the biological properties of these innovative metallodrugs.

## MATERIALS AND METHODS

### Drugs

**Figure 1:**
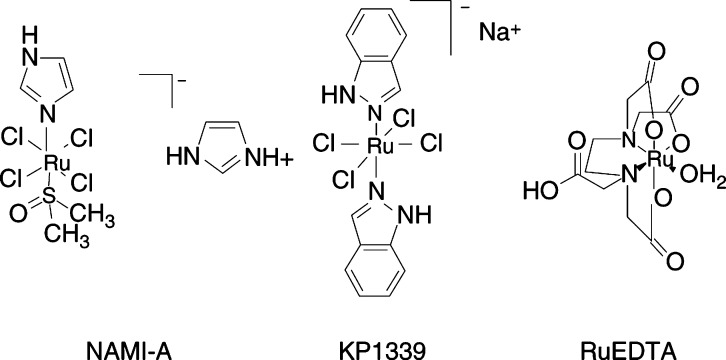
Schematic representation of the chemical structure of NAMI-A, KP1339 and RuEDTA as derived from crystallographic data. Notably, all complexes are characterised by the presence of at least one labile ligand.

NAMI-A was a gift of Professor E Alessio (Department of Chemical Sciences, University of Trieste), KP1339 (sodium *bis* indazole tetrachloro ruthenate, Na[*trans*-RuCl_4_Ind_2_]) was provided by Professor B Keppler (Department of Inorganic Chemistry, University of Vienna), and RuEDTA (K[Ru(HEDTA)Cl]) was prepared according to the published procedure (Diamantis and Dubrawsky, 1981). The identity and purity of the compounds was checked by various analytical techniques including elemental analysis, absorption and FT-IR spectroscopy. The chemical features of these ruthenium(III) complexes are shown in Figure 1

. RuEDTA is a classical ruthenium(III) aminopolycarboxylate complex whose chemical properties have been extensively documented in previous studies (Matsubara and Creutz, 1979; Diamantis and Dubrawsky, 1981); NAMI-A and KP1339 are mixed ligand ruthenium(III) complexes (Alessio *et al*, 1993; Kratz *et al*, 1994). All are characterised by the presence of one or more labile ligands that can be replaced by NO. All these ruthenium(III) complexes are soluble in aqueous physiological buffers. RuEDTA is sufficiently stable under physiological conditions except it slowly dimerises (Bottomley, 1978). Both NAMI-A and KP1339, when dissolved in water or in physiological buffers, progressively hydrolyse and release the ruthenium(III) bound chlorides giving rise to a number of hydrated and/or hydroxy ruthenium species, as described (Mestroni *et al*, 1994; Kung *et al*, 2001).

Different NONOates were used as source of NO, mainly MAHMA NONOate (*Z*-1-{*N*-methyl-*N*-[6-(*N*-methylammoniohexyl)amino]}diazen-1-ium-1,2-diolate, Alexis Biochemicals, Lausen, Switzerland).

Acetylcholine hydrochloride, noradrenaline [(−)arterenol hydrochloride], sodium nitroprusside (NaNP) and the cGMP stable analogue, 8-Br-cGMP, were purchased from Sigma (St Louis, MO, USA). Vascular endothelial growth factor (VEGF) was purchased form Peprotech (Calbiochem, Milan, Italy).

### NO-binding studies

Binding of NO to the individual ruthenium(III) complexes was analysed by different spectroscopic techniques including visible absorption, FT-IR and ^1^H-NMR. Visible spectrophotometric studies were carried out on a Perkin-Elmer Lambda 20 BIO instrument equipped with a thermostated cuvette. The hydrolysis of ruthenium(III) complexes and the NO binding processes were monitored continuously over 24 h, acquiring spectra at 10 min intervals. The experiments were carried out in phosphate buffer (NaH_2_PO_4_ 50 mM, NaCl 100 mM, pH 7.4) at 25°C, working at 100 *μ*M ruthenium(III) concentrations. FT-IR spectra of similar samples were recorded on a Perkin-Elmer Spectrum BXI instrument. 300 MHz ^1^H-NMR spectra were measured on a Gemini 2000 Varian Instrument. For ^1^H-NMR measurements, 1 mM samples were employed.

### Aortic ring relaxation

The thoracic segment of the aorta was obtained from male New Zealand rabbits and cut into rings of 3–4 mm width (four to six rings from each aorta). The preparations were suspended between stainless-steel hooks and mounted in a 10 ml organ bath filled with warmed (37°C) and oxygenated (95% O_2_, 5% CO_2_) Krebs solution. The incubation solution had the following composition (mM): NaCl 118, NaHCO_3_, 25, KCl 4.7, KH_2_PO_4_ 1.2, MgSO_4_ 1.2, CaCl_2_ 2.5, glucose 10. A tension of 2 **g** was applied and isometric contraction was recorded by a transducer on a polygraph chart (Battaglia Rangoni). After 60–90 min of equilibration, a concentration–response curve for noradrenaline (NA, 0.1–1 *μ*M) was performed and a concentration able to induce 50% of the maximum effect was chosen. Following wash-out, the rings were preconstricted with this concentration of NA and the cumulative relaxant effect of acetylcholine (ACh, 0.1–10 *μ*M) was tested. The value of tension developed following NA administration (1168±68 mg, *n*=7) was taken as 100% and the effect of ACh was referred to this value. The vasorelaxant effect of ACh was tested 10 min after the contractile response to NA was fully developed. Experiments in which 10 *μ*M ACh induced a maximum relaxant effect of less than 40% were discarded. The preparation was then washed, and a second concentration–relaxant effect curve for ACh was obtained in the same preparation preconstricted by NA in the presence of test compounds with or without 8-Br-cGMP (30 min exposure).

### Cell lines and culture conditions

Coronary venular endothelial cells (CVEC) were isolated and characterised as previously described (Morbidelli *et al*, 1996). Cells were maintained in culture in Dulbecco's modified Eagle's medium (DMEM) supplemented with 10% bovine calf serum (CS) and antibiotics (100 U ml^−1^ penicillin and 100 *μ*g ml^−1^ streptomycin) on gelatin-coated dishes. Cells were cloned and each clone was subcultured up to a maximum of 25 passages. Passages between 15 and 20 were used in these experiments.

### Cytotoxicity

The cytotoxic effect of test substances was studied by trypan blue exclusion. Briefly, endothelial cells were suspended in 0.1% CS medium supplemented with increasing concentrations of the test compounds and incubated at 37°C for 4 h. Cells were then counted in a haemocytometer and the percentage of dead cells over the total number of cells was calculated.

### Migration assay

Cell migration was assessed in 48-well microchemotaxis chambers (NeuroProbe, Biomap, Milan, Italy) on a polycarbonate filter (8 *μ*m pore size). The filter was coated with type I collagen (100 *μ*g ml^−1^) and bovine plasma fibronectin (10 *μ*g ml^−1^). A cell suspension containing 12 500 cells was added to the upper chamber of each well. Test compounds were added to the cells in the upper compartments, while angiogenic factors or NO donor drugs were placed in the lower wells. After for 4 h at 37°C, cells that had not migrated were removed and the filter was stained with Diff-Quik (Biomap, Milan, Italy). Migrated cells were counted in 10 random fields per well at a magnification of 400 (Ziche *et al*, 1997).

### Cell proliferation/survival

Cell proliferation and survival were quantified by total cell number as reported (Ziche *et al*, 1997). Briefly, 1.5 × 10^3^ cells resuspended in 10% CS were seeded in each well of 96 multiwell plates. After adherence (4 h) the medium was replaced with 1% CS medium containing test compounds in the absence or in the presence of the angiogenic factors VEGF or the NO donor drug NaNP, and incubated for 48 h. The supernatants were removed from the multiwell plates and the cells were fixed with methanol and stained with Diff-Quik. Cell numbers were obtained by counting seven random fields at a magnification of 100 with the aid of an ocular grid.

### Rabbit corneal angiogenesis assay

Corneal assays were performed in New Zealand white rabbits (Charles River, Calco, Como, Italy) in accordance with the guidelines of the European Economic Community for animal care and welfare (EEC Law No. 86/609). Briefly, after being anaesthetised with sodium pentothal (30 mg kg^−1^), slow-release pellets bearing test substances were implanted in micropockets surgically produced in the lower half of the cornea. Subsequent daily observation of the implants was made with a slit-lamp stereomicroscope without anaesthesia. An angiogenic response was scored positive when budding of vessels from the limbal plexus occurred after 3–4 days and capillaries progressed to reach the implanted pellet. The angiogenesis score was calculated (vessel density × distance from the limbus) as described (Ziche *et al*, 1997).

### Statistical analysis

Results are expressed as mean values±s.e.m. Multiple comparisons were performed by one-way ANOVA and individual differences tested by Fisher's test after the demonstration of significant intergroup differences by ANOVA.

## RESULTS

### Reaction of ruthenium(III) compounds with NO

Reactions of NAMI-A, KP1339 and RuEDTA with NO were carried out in the reference phosphate buffer and analysed by absorption and FT-IR spectroscopies and by ^1^H-NMR. MAHMA NONOate was used as the source of NO. Notably, each MAHMA molecule releases two NO equivalents. The molar ratio MAHMA/Ru(III) used in this study was 2 : 1.

In the case of RuEDTA, NO bound quickly to the ruthenium(III) centre blocking RuEDTA dimerisation, in agreement with previous results by Fricker (Davies *et al*, 1997; Fricker, 1999; Fricker *et al*, 1997). The main product of the reaction was the diamagnetic RuEDTA–NO adduct, characterised by an intense FT-IR band at 1880 cm^−1^.

**Figure 2:**
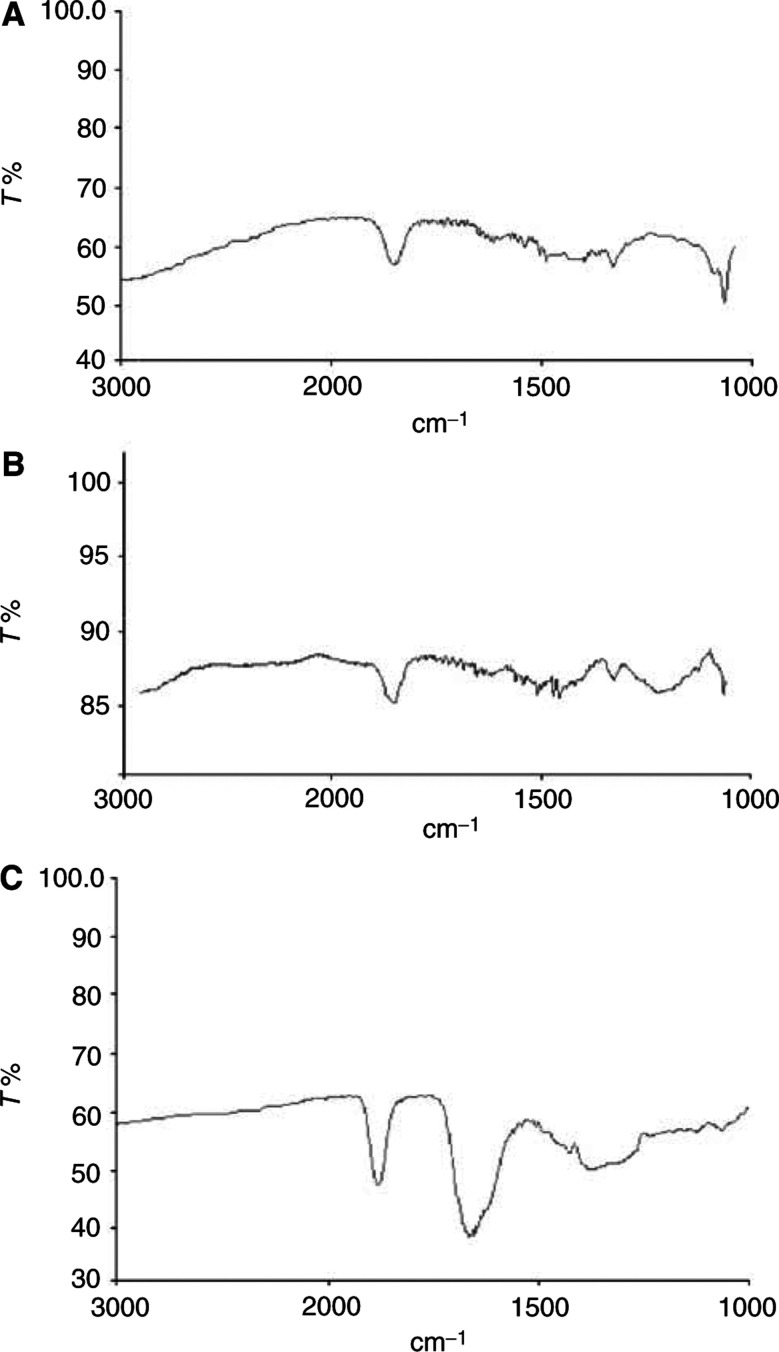
FT-IR spectra of the reaction products of NAMI-A (**A**), KP1339 (**B**) and RuEDTA (**C**) with NO. The intense band observed in the 1900–1800 cm^-1^ range is diagnostic of the presence of an Ru–NO moiety.

FT-IR spectra for NO adducts of either NAMI-A or KP 1339 are shown in Figure 2

. Intense transitions, at 1850 and 1840 cm^−1^ respectively, were observed indicating formation of the [Ru–NO] moiety. Independent NMR data showed that both paramagnetic ruthenium(III) complexes upon reaction with NO rapidly transformed into related diamagnetic species, formally containing the {Ru^II^ NO^+^} moiety (data not shown). For all complexes, the disappearance of the paramagnetic lines and the associated formation of the NO adducts was complete within a few minutes.

### Influence of NO scavengers on ACh-induced relaxation in aortic rings

The pharmacological characterisation of the above compounds was performed in rabbit aortic ring preparations bearing intact endothelium.

**Figure 3:**
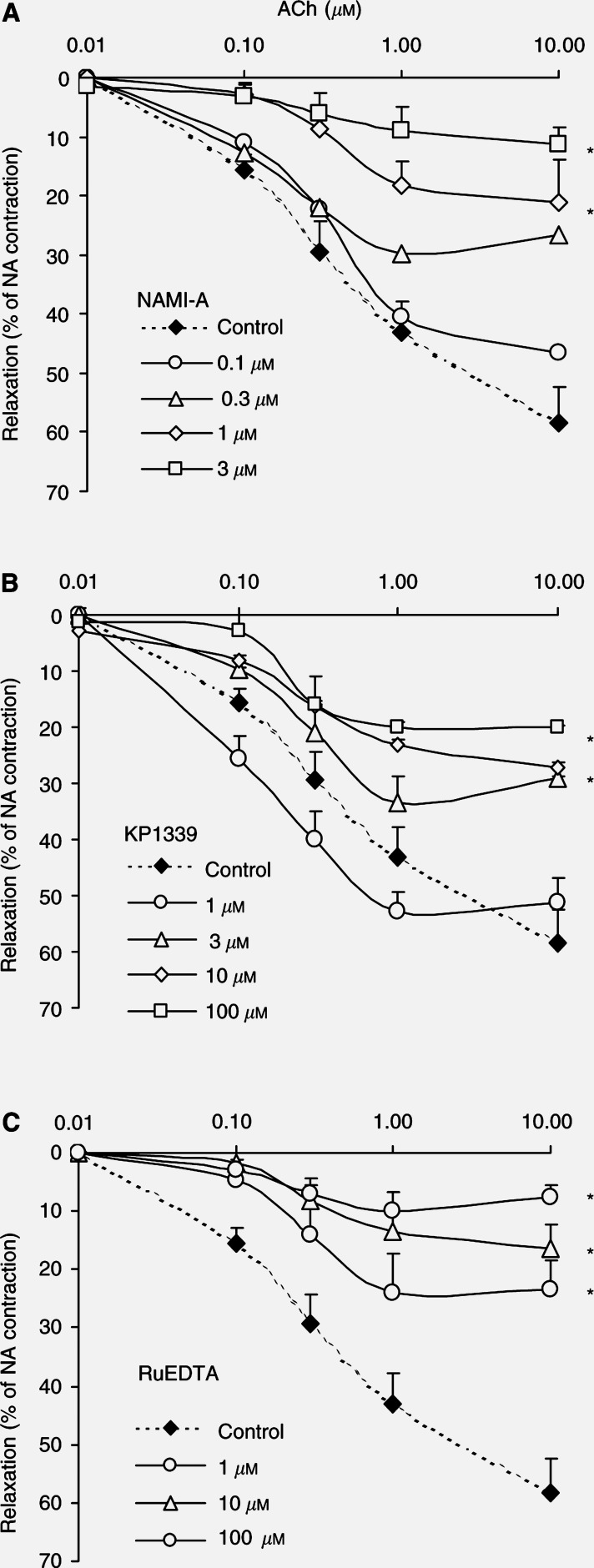
Vasodilator responses of aortic ring preparations by increasing concentrations of ACh. Noradrenaline (NA)-preconstricted rabbit aortic rings were exposed to Krebs solution in the absence and in the presence of increasing concentrations (0.1–3 *μ*M) of NAMI-A (**A**), (1–100 *μ*M) of KP1339 (**B**), and (1–100 *μ*M) of RuEDTA (**C**) before stimulation with ACh (0.1–10 *μ*M). Vasodilatation is compared to ACh-induced responses. Data are means±s.e.m. of five ring preparations. ^*^P<0.05 *vs* basal.

Vessel preparations were incubated for 30 min at 37°C with increasing concentrations of the compounds (0.1–3 *μ*M for NAMI-A, 1–100 *μ*M for KP1339 and 1–100 *μ*M for RuEDTA) before challenging with ACh. NAMI-A, KP1339 and RuEDTA were able to interfere to a variable extent with ACh-induced vasorelaxation in a concentration-dependent manner (Figure 3

). The rank order of potency was the following: NAMI-A>RuEDTA>KP1339 with EC_50_s for 1 *μ*M ACh-induced relaxation of approximately 0.3, 1 and 10 *μ*M, respectively. The maximum effect caused by 10 *μ*M ACh was reduced by about 90% in the presence of 3 *μ*M NAMI-A and by 80% in the presence of 100 *μ*M RuEDTA. Not only the extent of vasorelaxation was reduced by NO scavengers but also the time required to obtain it. In the presence of NAMI-A, KP1339 and RuEDTA, ACh-induced vasorelaxation was obtained within 60–90, 90–120 and 90–120 s, respectively, *vs* 35–40 s in the absence of drugs.

We then tested whether the molecular mechanism of these NO scavengers was because of inhibition of NO biosynthesis. Since cyclic GMP (cGMP) and protein kinase G (PKG) are the intracellular effectors of the vasodilatory effect of NO, we checked whether the addition of 8-Br-cGMP, a stable cell-permeable analogue of cGMP, could revert the vasoconstrictive effect of these NO scavengers. In the presence of 1 *μ*M NAMI-A, 3 *μ*M 8-Br-cGMP was able to restore the vasodilatory effect of ACh by 66 % (data not shown).

### Effect of NO scavengers on endothelial cell migration

**Figure 4:**
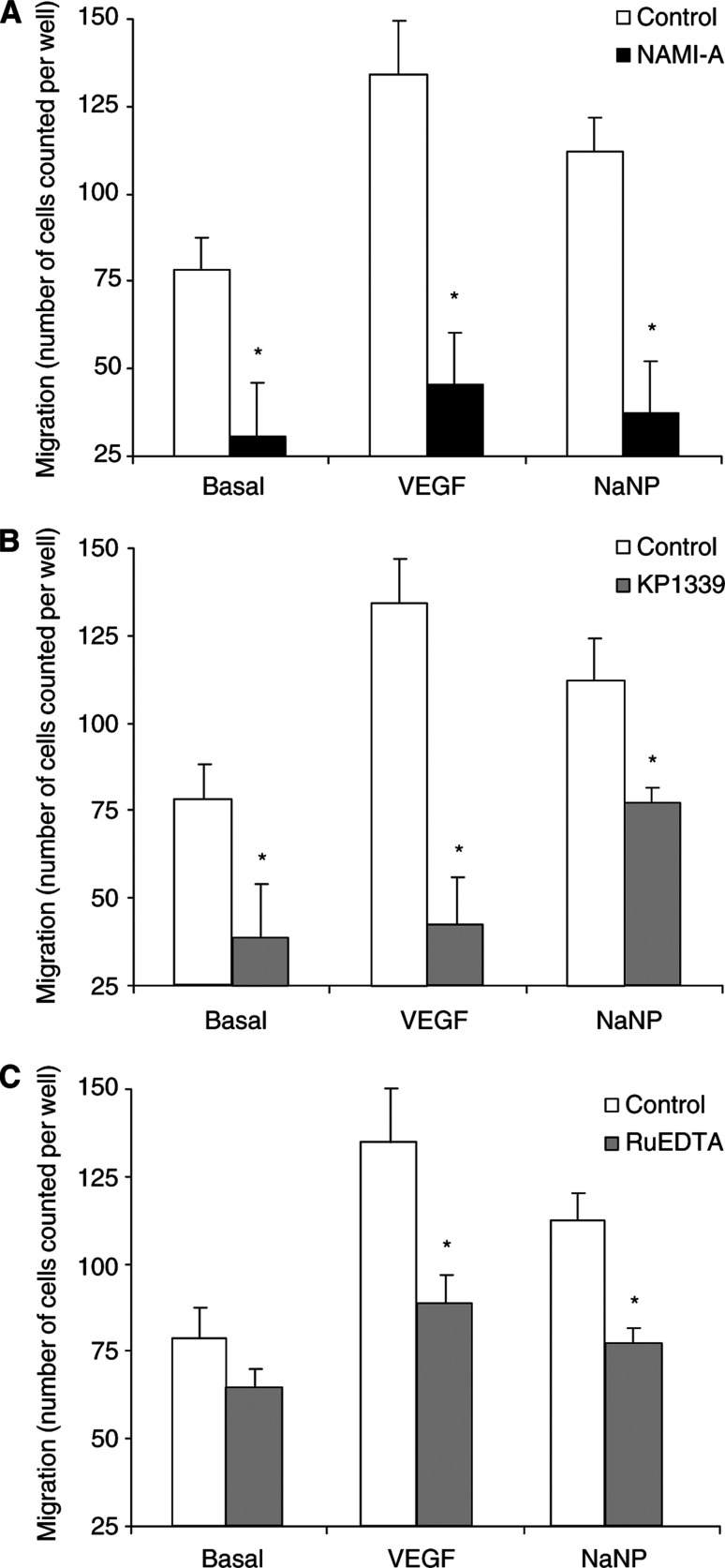
Migration of postcapillary endothelial cells towards VEGF or exogenous NO. Following incubation with NO scavengers (3 *μ*M NAMI-A, 3 *μ*M KP1339 or 30 *μ*M RuEDTA) endothelial cells were challenged towards gradients of VEGF or NaNP in microchemotaxis Boyden chambers. After 4 h, cell migration was measured by microscopic examination. Data (means±s.e.m.) are reported as the number of cells counted per well (*n*=3 in triplicate). ^*^P<0.05 *vs* control.

Microvascular endothelial cell migration and proliferation are key events of the angiogenesis process. As previously demonstrated, VEGF and NaNP are able to induce endothelial cell migration. The effect of VEGF is mediated by the activation of endothelial-constitutive NOS, NO production and cGMP accumulation (Morbidelli *et al*, 1996; Ziche *et al*, 1997). Endothelial cells exposed to NO scavengers were thus challenged toward a gradient of VEGF (20 ng ml^−1^) or NaNP (10 *μ*M). Cells pretreated with NAMI-A or KP1339 (3 *μ*M each) or RuEDTA (30 *μ*M) showed reduced numbers of migrating cells in the basal condition, and these compounds were able to block completely the migratory effect elicited by VEGF or NaNP (Figure 4

). KP1339 was the least effective in reducing NaNP-induced migration, in accordance with the pharmacological data on ACh-induced vasorelaxation.

The inhibitory effect of metallodrugs was not because of cytotoxicity, since no statistically relevant toxic effect was observed in endothelial cell suspensions exposed to the NO scavengers for 4 h at 37°C (Table 1

**Table 1:** Lack of cytotoxic effect of NO scavengers on endothelial cells

Concentration (*μ*M)	NAMI-A	KP1339	Ru-EDTA
0	6±3	6±3	6±3
1	15±5	6±1	ND
3	13±4	8±1	ND
10	15±5	8±5	9±5
100	9±1	11±1	12±3

Cytotoxicity of compounds was studied in endothelial cells by trypan blue exclusion after 4 h of incubation at 37°C. Data (means±s.e.m.) are reported as the percentage of dead cells over the total number of cells counted. ND=not determined.

).

### Effect of NO scavengers on endothelial cell proliferation

**Figure 5:**
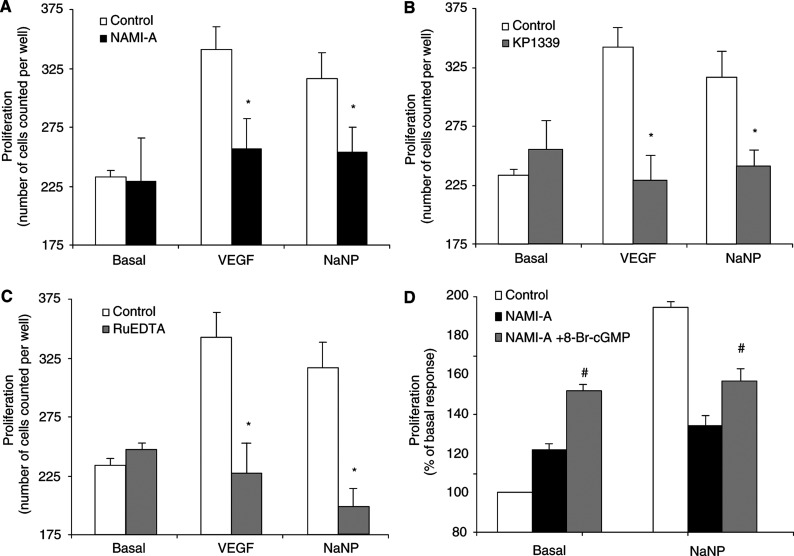
Proliferation of postcapillary endothelial cells in the presence of VEGF or exogenous NO. (**A**–**C**) Cells were incubated in the absence and in the presence of NO scavengers (3 *μ*M NAMI-A, 3 *μ*M KP1339 or 30 *μ*M RuEDTA). Cell proliferation was monitored after 48 h incubation. Data (means±s.e.m.) are reported as number of cells counted per well. (**D**) Cell proliferation in response to 10 *μ*M NaNP was studied in endothelial cells exposed to 3 *μ*M NAMI-A in the presence of 100 *μ*M 8-Br-cGMP (*n*=3 in triplicate). ^*^P<0.05 *vs* control and ^#^*P*<0.05 *vs* NAMI-A alone.

Endothelial cell proliferation is potently stimulated by either VEGF or exogenous NO, the former acting through NOS and MAPK pathway (Morbidelli *et al*, 1996; Parenti *et al*, 1998). As shown in Figure 5(A–C)

, endothelial cell proliferation induced by VEGF (20 ng ml^−1^) or NaNP (10 *μ*M) was markedly inhibited by all NO scavengers (3 *μ*M NAMI-A or KP1339 or 30 *μ*M RuEDTA). The blockade of cell growth was accompanied by a change in their morphology with the appearance of cytoplasmic vacuoles (data not shown).

When cells were incubated with 3 *μ*M NAMI-A in the presence of 100 *μ*M 8-Br-cGMP, the proliferative effect of NaNP and VEGF was restored (Figure 5D), demonstrating that the molecular mechanism of NAMI-A in interfering with endothelial cell growth was the blockade of soluble NO.

### Effect of NO scavengers on *in vivo* angiogenesis

**Figure 6:**
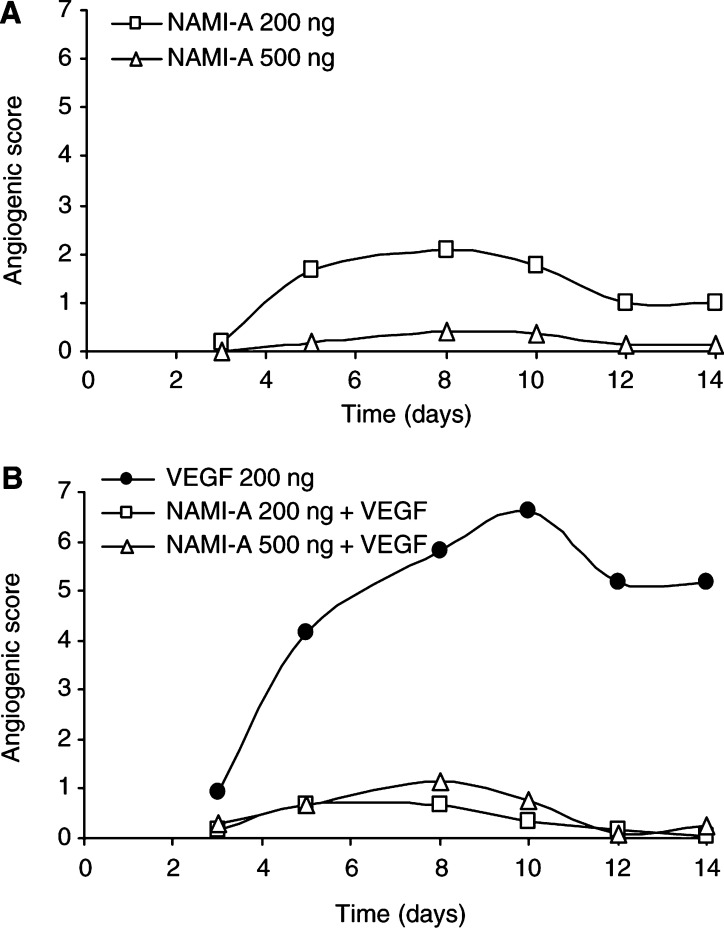
Effect of NAMI-A on *in vivo* angiogenesis in the rabbit corneal model. Pellets bearing NAMI-A and/or VEGF165 were prepared and surgically implanted in the corneal stroma of albino rabbits. The angiogenic response of NAMI-A was tested *per se* (**A**) and in the presence of VEGF (**B**). Data are reported as angiogenic score during time (days). Numbers are means of at least three implants per experimental point.

The antiangiogenic activity of NAMI-A observed in isolated cells was also expressed *in vivo* in the avascular rabbit cornea against the strong angiogenic response elicited by VEGF. Slow-release pellets were prepared incorporating two different doses of NAMI-A, namely 200 and 500 ng, alone and in the presence of 200 ng VEGF. NAMI-A did not affect angiogenesis ‘*per se*’ and did not induce any inflammatory response. The compound was able to inhibit VEGF-induced response completely (Figure 6

).

## DISCUSSION

Ruthenium(III) complexes are an emerging family of metallodrugs that are finding application as potential agents for the treatment of cancer, septic shock, immune diseases and other pathological conditions (Clarke *et al*, 1999). Among these agents, NAMI-A, a compound found to be active against lung metastases (Sava and Bergamo, 1999), tumour cell invasion (Zorzet *et al*, 2000), and recently to possess antiangiogenesis properties (Pintus *et al*, 2002; Sanna *et al*, 2002; Vacca *et al*, 2002) is presently undergoing clinical trials as an antimetastatic agent.

Three representative ruthenium(III) complexes, namely NAMI-A, KP1339 and RuEDTA were selected for further investigation in an attempt to relate the NO scavenging properties to their activity on the angiogenesis process. Unambiguous evidence for the formation of tight ruthenium–nitrosyl adducts, and therefore NO scavenging properties are here reported for NAMI-A and KP1339. The properties of RuEDTA, previously reported (Davies *et al*, 1997), have been confirmed.

Notably, these three complexes, upon reaction with NO, even at stoichiometric ratios, exhibit a spectral behaviour that is diagnostic of the formation of stable ruthenium–nitrosyl species, that is the appearance of an intense IR transition at 1900–1800 cm^−1^ and the disappearance of the hyperfine signals in the ^1^H-NMR spectra owing to loss of paramagnetism. In all cases the reaction is complete within a few minutes.

The relaxation induced in rabbit isolated vessels by ACh, an experimental paradigm originally employed for revealing the role of NO in mediating blood vessel relaxation (Furchgott and Zawadzki, 1980), is inhibited by all ruthenium complexes examined, demonstrating their ability to interfere with NO binding also in biological systems. NAMI-A is the most potent, whereas KP1339 the weakest agent in antagonising the NO-mediated effects. Reversal of the inhibition by 8-Br-cGMP, the intracellular NO effector, clearly indicates that these complexes prevent NO from exerting its action without affecting the intracellular relaxation mechanism.

Endothelial cell functions linked to angiogenesis such as migration and proliferation, typically stimulated by exogenous NO donors (NaNP) or VEGF, the latter leading to sequential activation of NOS and of the GMP-dependent kinase cascade (Morbidelli *et al*, 1996; Ziche *et al*, 1997; Parenti *et al*, 1998), are exquisitely sensitive to ruthenium complexes. All compounds reduce, to a different extent, the migration of endothelial cells. Similarly, the ruthenium complexes affect the stimulated proliferation of endothelial cells measured at 48 h, indicating their long-lasting effects as NO scavengers. Since 8-Br-cGMP counteracts the inhibition exerted by ruthenium compounds on the proliferative effects elicited by NaNP or VEGF, it appears that the action of these compounds is again exclusively confined to the capture of nascent NO, without affecting the intracellular mechanisms involved in proliferation. The absence of cell toxicity further support the notion that these compounds, at the concentrations examined, have no major intracellular effects. The efficacy of NAMI-A as a NO scavenger, observed in *in vitro* conditions driven by an increased availability of NO, is confirmed *in vivo* in the rabbit cornea assay. The compound exerts strong inhibition towards VEGF, whose angiogenic response has been shown to be dependent on the activation of the NOS pathway (Ziche *et al*, 1997; 2000).

The results of this study confirm recent findings on NAMI-A demonstrating its inhibitory activity on proliferation, cell migration and production of degradative enzymes (Vacca *et al*, 2002; Sanna *et al*, 2002). Also the antiangiogenic properties previously observed in the chorioallantoic membrane (Vacca *et al*, 2002) are confirmed in our *in vivo* assay in the rabbit cornea.

The inhibition of angiogenesis exerted by NAMI-A has been attributed to induction of apoptosis, which in turn is linked to inhibition of the mitogen-activated protein kinase (MAPK) signalling pathway and heat shock protein-27 downregulation (Pintus *et al*, 2002; Sanna *et al*, 2002). Since MAPK is a downstream effector of the NOS/cGMP pathway (Parenti *et al*, 1998), its inhibition by NAMI-A may be caused by the NO-binding activity here reported. In support of this interpretation, involving NO as a crucial signalling molecule, is the finding that a cGMP stable analogue is able to revert the antiangiogenic effect of NAMI-A.

In conclusion, this study demonstrates that ruthenium(III) compounds inhibit NO-dependent angiogenesis, and highlights a rather innovative mechanism of action for heavy metal-based compounds, which are currently hypothesised to act via DNA-binding (Malina *et al*, 2001). The antimetastatic activity of these metallodrugs might be multiple, interfering with the endothelial cell functions during angiogenesis, angiogenic factor overexpression, the vasodilating state of tumours and probably tumour cell invasiveness, each event being demonstrated by different groups as NO-dependent (Fukumura *et al*, 1997; Gallo *et al*, 1998; Jadeski and Lala, 1999; Orucevic *et al*, 1999; Jadeski *et al*, 2000; Morbidelli *et al*, 2001; Feng *et al*, 2002). On the speculative side, it may be suggested that tumours producing high NO levels and exhibiting a high angiogenic output would be more sensitive to ruthenium(III)-based drugs.
